# Supplementary material for: Retinal oxygen saturation changes progressively over time in diabetic retinopathy
Source: PLoS One. 2021 May 12;16(5):e0251607. doi: 10.1371/journal.pone.0251607 (PMC8115819; doi:10.1371/journal.pone.0251607)
Supplement: Attachment — Submitted filename: Hardarson Response to reviewers.docx [file pone.0251607.s001.docx]

Dear academic editor and reviewers

We greatly appreciate the comments made on our manuscript (PONE-D-21-04724, Retinal oxygen saturation changes progressively over time in diabetic retinopathy)

Below is our response. We have also included a marked copy of the manuscript, where changes are marked, as well as an unmarked version of the updated manuscript.

**Replies to the academic editor**

*The reviewers indicate that your study is worthwhile and presented well, but they list numerous minor shortcomings that should be addressable by modifying the text.  For example, if you analyzed only one eye per patient, how did you select that eye? What was the rate of progression in patients excluded because of treatment? Was the retinal grader aware of the grade assigned by the nurse grader?*

Reply:

Thank you. We have addressed these comments and other comments raised by the reviewers. In short:

- Only the right eye was selected and this has been clarified in the manuscript.
- We excluded those who had ocular treatment during follow-up. This was done to avoid confounding effects of treatment, i.e. to isolate the effects of disease progression. This selection means that progression in our sample is decreased. We have added discussion on this in the manuscript.
- The retinal grader was not aware of the grading by the nurse grader. This has been clarified in the manuscript.

The above points are further addressed in replies to the reviewers below.

**Journal Requirements**

(Below, we address the requirements that specifically needed to be addressed for this manuscript)

*Any changes to the reference list should be mentioned in the rebuttal letter that accompanies your revised manuscript.*

We have added the following references (see also replies to the reviewers, below).

*20.	Felder AE, Wanek J, Blair NP, Joslin CE, Brewer KC, Chau FY, et al. The Effects of Diabetic Retinopathy Stage and Light Flicker on Inner Retinal Oxygen Extraction Fraction. Invest Ophthalmol Vis Sci. 2016;57(13):5586-92. doi: 10.1167/iovs.16-20048. PubMed PMID: 27768785.*

*21.	Blair NP, Wanek J, Felder AE, Joslin CE, Kresovich JK, Lim JI, et al. Retinal Oximetry and Vessel Diameter Measurements With a Commercially Available Scanning Laser Ophthalmoscope in Diabetic Retinopathy. Invest Ophthalmol Vis Sci. 2017;58(12):5556-63. doi: 10.1167/iovs.17-21934. PubMed PMID: 29079858; PubMed Central PMCID: PMCPMC5656420.*

*26.	Teng PY, Wanek J, Blair NP, Shahidi M. Inner retinal oxygen extraction fraction in rat. Invest Ophthalmol Vis Sci. 2013;54(1):647-51. Epub 2013/01/10. doi: 10.1167/iovs.12-11305. PubMed PMID: 23299486; PubMed Central PMCID: PMCPMC3558991.*

*30.	Mehlsen J, Erlandsen M, Poulsen PL, Bek T. Individualized optimization of the screening interval for diabetic retinopathy: a new model. Acta Ophthalmol. 2012;90(2):109-14. doi: 10.1111/j.1755-3768.2010.01882.x. PubMed PMID: 20384605.*

*31.	Mehlsen J, Erlandsen M, Poulsen PL, Bek T. Identification of independent risk factors for the development of diabetic retinopathy requiring treatment. Acta Ophthalmol. 2011;89(6):515-21. Epub 2009/11/17. doi: 10.1111/j.1755-3768.2009.01742.x. PubMed PMID: 19912134.*

*38.	Vergmann AS, Torp TL, Kawasaki R, Hestoy DH, Wong TY, Peto T, et al. Retinal vascular oxygen saturation in response to a less extensive laser treatment in proliferative diabetic retinopathy. Acta Ophthalmol. 2020. Epub 2020/12/24. doi: 10.1111/aos.14727. PubMed PMID: 33354935.*

*Please provide additional details regarding participant consent. In the ethics statement in the Methods and online submission information, please ensure that you have specified what type you obtained (for instance, written or verbal, and if verbal, how it was documented and witnessed).*

We have added to the Methods section that the informed consent was in written form.

*Thank you for stating the following in the Competing Interests section:*

*"Sveinn Hakon Hardarson and Einar Stefánsson have commercial interest in the company Oxymap ehf. They have stock in the company, are on its board and are listed on two patents related to retinal oximetry (Automatic registration of images US 7774036 B2, Temporal oximeter WO 2010143208 A3)."*

*Please confirm that this does not alter your adherence to all PLOS ONE policies on sharing data and materials, by including the following statement: "This does not alter our adherence to  PLOS ONE policies on sharing data and materials.”*

*Please include your updated Competing Interests statement in your cover letter; we will change the online submission form on your behalf.*

We confirm that the commercial interest does not alter our adherence to PLOS ONE policies on sharing data and materials. We will include the updated competing interests statement in our cover letter.

*Please ensure that you refer to Figures 1 and 2 in your text as, if accepted, production will need this reference to link the reader to the figure.*

We have made sure that this is the case.

**Responses to reviewer 1**

*Thank you for this interesting paper about the relationship between retinal oxygen saturation, DR level and red lesion count. It is a well conducted study with important results. However, I have some small corrections/suggestions to make the paper and especially the discussion section more in-depth and thorough. These are noted below.

Introduction:
Page 4, line 61-63: Can you please elaborate shortly why this would be desirable?*

This is a good suggestion. We have added the following:

*„Monitoring metabolic changes, which are likely to occur early, may provide an opportunity for more timely management of the disease.“*

Methods:
Page 7-8, line 127-128: Was the retinal grader aware of the degree determined by the nurse grader when grading images? If yes, this could be subject to some bias. Please clarify this. Furthermore, add a section in the discussion section if this is the case.

The retinal grader doing the oximetry analyses was not aware of the grading done by the nurse grader. We have clarified this in the Methods section and added the highlighted text:

*„At the time of the study, oximetry images from all follow-up examinations were anonymized and transferred to the University of Iceland, where the analysis was performed masked to grading of patients and other clinical data and the identity of the patients.“*

*Discussion:
Page 14, line 256-257: You may have a median follow up length of three years. However, there were a broad range in FU time. Please discuss what impact the difference in follow up length could have had on the results.*

In our opinion, variable follow-up time is both a strength and a weakness. Longer follow-up in general would probably have allowed us to see more definite progression in structural parameters. However, with variable follow-up time we could see indications that oximetry values seem to change gradually over a rather short follow-up time while the structural parameters change less. We have added the following:

*„Some patients had a very short follow-up time and others had longer. This variable follow-up time allowed us to test whether changes in oximetry values or structural parameters are correlated with length of follow-up.“*

*Page 15, line 278-280: How can you conclude this? Increase in oximetry is not a validated method for assessing progression of DR. Though it increased with time it is not completely clear that degree of DR does as well just because time has passed. It might be a stretch to conclude this. I suggest you rephrase, thank you.*

This is true. We have rephrased in the following way (we added the highlighted part and erased another part):

*„Oxygen saturation, however, clearly increased with length of follow-up in both arterioles and venules. The changes in saturation with time may reflect underlying metabolic progression of the disease that may later manifest more clearly in morphological progression of diabetic retinopathy. This will be tested with longer follow-up. It must be kept in mind, that most patients had retinopathy at baseline and it remains to be seen whether a change in oxygen saturation precedes the initiation of diabetic retinopathy.“*

*Page 16, line 317: I suggest you also add a recently published paper in ACTA by Vergmann et al. (PMID: 33354935) that also involves this subject.*

Thank you for pointing this out. We have added the reference.

*There was low quality in some images (down to 3.5) – is it possible to conduct proper analysis of images with that poor quality? Please comment on this, thank you.*

It is true that this is very low quality and such measurements are likely flawed. Only two images at the first visit and two at the second visit had image quality lower than 5.0. These images are unlikely to have affected the combined analyses of 214 patients. We could have set a cut-off value for image quality. Instead we decided to include all images that could be measured (even if the measurements were crude in a few cases). We then tested if image quality changed between the first and second session. On average, the image quality grade decreased slightly, from 7.7 to 7.4. This is included in the manuscript.

Our main worry is always that we are mislead in our conclusions on oxygenation by changes or differences in image quality. In this case, we do not have to worry much since a decrease in image quality has been shown to artificially decrease measured saturation (ARVO abstract: Invest. Ophthalmol. Vis. Sci. 2015;56(7 ):3316). Since we see an increase in measured saturation from first to second visit, it is very unlikely that image quality has affected our main conclusions (slightly lower image quality at the second visit may have slightly attenuated the change found in saturation but not inflated it or created it).

*What are your thoughts about the fact that you did not find any patients with level 3 or 4 DR? Please discuss this.*

This is mostly a reflection of the nature of the screening programme. In the general screening population in Denmark, not so many patients - fortunately - have advanced degrees of diabetic retinopathy. Also, those with ocular treatment were excluded. We have added the following to the discussion section:

*„Nevertheless, there was little progression, which is likely due to the intensive control by endocrinologists for these patients. and due to exclusion of patients who received ocular treatment between visits.“*

*You mention a lot of strengths of oximetry. I suggest you also mention some of the limitations with this method.*

This is a good point. We have added the following paragraph to the discussion section:

*„The main strengths of retinal oximetry are its non-invasive nature and the ability to gauge metabolism. There are also limitations, which include possible effects of image quality on results and a relatively small size of change in individual patients. It is most likely that retinal oximetry can be used with other parameters, rather than as a standalone measurement. The same is true for almost every parameter in management of diabetes and diabetic retinopathy.“*

**Responses to reviewer 2**

*722 consecutive patients with diabetes underwent ocular examination including retinal examination, fundus photography and retinal oximetry with the Oxymap T1 retinal oximeter. The latter provides vascular diameter and oxygen saturation measurements. The examination resulted in treatment in 294 of these. Of the remaining 428 patients, 216 could not be followed-up. 214 patients were followed up for a median time of 3.0 years. The authors’ purpose was to relate the oximetry results to clinical diabetic retinopathy grades (as per Wilkinson, et al) and fundus red lesion counts. The authors rightly note the limitations of assessing diabetic retinopathy in terms of visible retinal lesions (Table 2 shows 83% patients had no change in clinical stage over the time studied). They also rightly show the need for physiologically based metrics, especially in when established longitudinally, as in the current manuscript.*

*The manuscript treats the results by patient, not eye. Apparently, only one eye was included in the study per patient. The manuscript should indicate how the eye included was chosen, eg all right eyes, the most severe eye, by randomization, etc.*

We used only right eyes, to keep analyses simple and robust. This was only mentioned in the abstract but we have now added the following to methods section. Thank you for pointing this out.

*„The right eye of each patient was measured“*

*All patients had stage 0 to 2 (moderate nonproliferative retinopathy) and none progressed to stage 3 or higher. With a cohort this large over the time indicated, it would be surprising if none progressed to stages higher than stage 2. This lack of progression should be explained explicitly. Was it that as soon as that happened they were treated and excluded from the untreated cohort? Though not stated in the paper, it seems likely that any patient with CSME was treated and thus was excluded from the untreated cohort. It would be best to state whether any eyes developed CSME after the initial examination and, thus, were treated and then excluded.*

This was not clear enough in the original manuscript and we have now added the following to the Methods section:

*„In 294 of these patients, the examination resulted in treatment of diabetic retinopathy and these patients were excluded from the study.“*

The follwing was also added to the Discussion section:

*„Nevertheless, there was little progression, which is likely due to the intensive control by endocrinologists for these patients and due to exclusion of patients who received ocular treatment between visits.“*

*It appears that gender and type of diabetes were not equally distributed, and it would be best to provide a statistical analysis to confirm this or disprove it. This would be mainly to be complete, since these factors are not likely to alter the results.*

*No statement on race is given. Elevation of retinal arterial SO2 in Hispanic patients was found in one paper (PMID: 29079858). It may well be that this cohort from Denmark had very few non-Caucasian patients, but that should be specified in the manuscript.*

It is true that there are more males and more type 2 patients. Both facts are likely to reflect the study population to some extent. All statistical tests focus on the change from the first to second visit and the underlying assumption is that on average, diabetic retinopathy progresses over time regardless of diabetes type or gender. The rate of progression could of course be somewhat different but we feel that our data is not appropriate for a thorough analysis of possible effects of diabetes type or gender.

Unfortunately, we do not have exact data on race. Most of the patients are of Caucasian and a few are of Middle Eastern origin. There are few if any of Hispanic origin.

We have added the following to the discussion on the limitations of the study (the first sentence was in the original manuscript but is included here for context):

*The selection process may mean that the studied sample is not entirely representative of progression of diabetic retinopathy in general. About two thirds of the patients were males and about two thirds of the group had type 2 diabetes. Most of the patients were of Caucasian origin.* *Importantly, we chose to exclude patients, who received ocular treatment between visits. This was done to isolate the effect of the disease progression, without confounding effects of different treatment. This probably also means that our selected patients had less progression than the study population as a whole.*

*The key findings in the cohort were little change in clinical grade, no significant change in red lesions, increases in arterial and venous SO2, and decrease in arteriovenous SO2 over time. The latter indicates a greater increase in venous than arterial SO2. It would be of interest to report the arteriovenous SO2 difference normalized by the arterial SO2. This also equals the inner retinal oxygen extraction fraction. The largest oximetry change was in venous SO2 at 1.74 %/year. For oximetry to serve as a useful clinical tool to follow diabetic retinopathy, it would seem that measurements over a period of at least a year would be required.*

We agree that the oxygen extraction fraction is an interesting metric. Below are the results for the difference in oxygen extraction fraction between visits compared to follow-up. As can be seen, the conclusion is very similar as for the arteriovenous difference, i.e. both the oxygen extraction fraction and the arteriovenous difference decreased with follow-up time.

*Figure above: Slope = -0.013, p<0.0001*

The conclusion for the relationship with change in red lesions is also the same, i.e. there is no relationship was found between change in oxygen extraction fraction and change in red lesions, p=0.67 (as is the case for AV difference vs red lesions).

We have added the following in the results section. However, we suggest limiting the addition to this short sentence since adding oxygen extraction fraction to all analyses would complicate the manuscript and in general lead to similar conclusions as using AV difference in this study.

*„Inner retinal oxygen extraction fraction, which is arteriovenous difference normalized by arteriolar saturation [ref. Teng et. al], also decreased significantly with time (p<0.0001).“*

1.74 percentage points‘ change during one year is, in our opinion, a rather large physiological change in short time. However, it is true that such a change could be difficult to measure reliably (and is sometimes less and sometimes more, depending on patients). We think it is most likely that retinal oximetry could be one of several variables used together when managing diabetic retinopathy. This is shortly mentioned in the manuscript.

*A considerable limitation of the work is that a large number of patients could not be followed-up. Whether these progressed at the same rate as found in the cohort cannot be known. As someone who has participated in several longitudinal studies, this reviewer knows how difficult it is to minimize patient losses to follow-up. Still, this loss to follow-up should be cited as a limitation.*

This is true. Following is the revised text on limitations due to the selection of patients:

*„The selection process may mean that the studied sample is not entirely representative of progression of diabetic retinopathy in general. About two thirds of the patients were males and about two thirds of the group had type 2 diabetes. Most of the patients were of Caucasian origin. Importantly, we chose to exclude patients, who received ocular treatment between visits. This was done to isolate the effect of the disease progression, without confounding effects of different treatment. This probably also means that our selected patients had less progression than the study population as a whole.“*

In summary, our study is not a good study of the general, natural history of diabetic retinopathy in the population. However, it highlights the difference between metabolic and structural changes in a subpopulation and suggests that further studies and longer follow-up should be attempted, possibly including also patients requiring treatment.

*Of particular interest would be the rate of progression in the cases that were excluded because of treatment. As the paper reads, most of these were excluded at the first examination. However, there may have been some patients who progressed to treatable disease after having a few oximetry exams. This reviewer suspects that they may have been progressing faster than those in the cohort in the manuscript. If there were such patients, it would be of interest to include them. On the other hand, the authors may be collecting such cases for a future manuscript focusing on this group.*

This is true. We plan to test the effects of further progression in future studies. However, one most be careful to avoid the confounding effects of ocular treatment inbetween the oximetry measurements. The selection process / study design is both a strength and a weakness in our opinion. The strength of our approach is that we get paired data, showing only the effects of disease progression (without the effects of ocular treatment) and the limited progression reveals that oximetry data and structural data behave differently.

*This reviewer notes that none of the papers from the Shahidi laboratory that have arterial and venous SO2 results in diabetic patients was cited in the Introduction. Inclusion of PMID: 29079858 and PMID: 27768785 is suggested.*

We apologize for this strange mistake on our part. We have gladly included references to these papers.
